# Quantifying Epigenetic Changes Induced by Chemical Exposure Using the epi-TK Assay

**DOI:** 10.21769/BioProtoc.5671

**Published:** 2026-04-20

**Authors:** Shiro Kuroki, Haruto Yamada, Mizuki Odagiri, Kei-Ichi Sugiyama, Manabu Yasui, Akira Sassa

**Affiliations:** 1 Department of Biology, Graduate School of Science, Chiba University, Chiba, Japan; 2 Division of Genome Safety Science, National Institute of Health Sciences, Tonomachi, Kawasaki-ku, Kawasaki-shi, Kanagawa, Japan

**Keywords:** Toxicology, Epi-genotoxicity, Reporter assay, TK6, *TK* gene, DNMT inhibitor

## Abstract

Epigenetic modifications play essential roles in regulating gene expression and maintaining cellular identity. Accumulating evidence suggests that chemical agents can contribute to carcinogenesis through epigenetic alterations, such as changes in DNA methylation and histone modifications, even in the absence of direct DNA damage. Here, we have developed a simple, cost-effective, and quantitative reporter assay, termed the epi-TK assay, to evaluate chemically induced epigenetic alterations. The assay is built upon the thymidine kinase (*TK*) gene mutation assay, a standardized and widely used in vitro genotoxicity assay for chemical safety evaluation. This system is based on an engineered human lymphoblastoid cell line (mTK6), in which the promoter region of the endogenous housekeeping *TK* gene is site-specifically methylated using epigenome-editing technology, resulting in stable transcriptional repression. Following chemical exposure, epigenetic perturbations at the *TK* locus are detected by culturing cells under hypoxanthine–aminopterin–thymidine selection and quantifying the frequency of TK revertant colonies, which reflects restoration of *TK* gene expression. Using the DNA methyltransferase 1 inhibitor GSK3484862 as a model compound, this protocol demonstrates that the epi-TK assay enables sensitive and quantitative detection of epigenetic state transitions. Importantly, this assay allows bi-directional detection of epigenetic changes, including DNA demethylation events and broader alterations in histone modification landscapes. Together, the epi-TK assay provides a practical and quantitative platform for evaluating epigenetic toxicity, with potential applications in chemical safety assessment frameworks.

Key features

• This protocol describes testing of the epigenetic effects of chemicals using the mTK6 cell line and a modified version of the *TK* gene-mutation assay.

• By employing a DNA-methylated housekeeping *TK* gene and colony formation as the readout, the assay enables quantitative epigenetic changes without the need for specialized equipment.

• The protocol offers a simple, quantitative, and cost-effective platform that is suitable for routine testing and comparative assessment of multiple compounds.

## Graphical overview


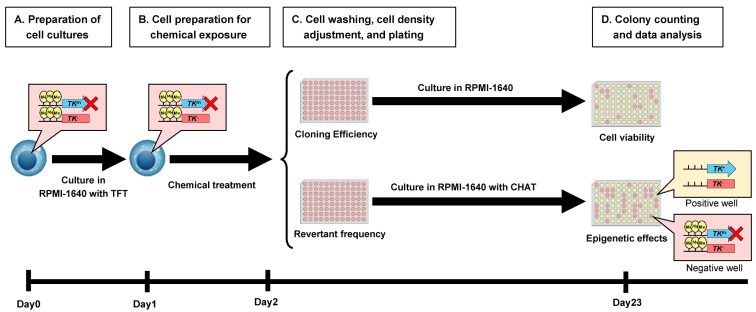


**Experimental flow of epi-TK assay.** The parental cell line TK6 is heterozygous at the *TK* gene locus, carrying one functional (*TK* ^+^) allele and one non-functional (*TK*-) allele. In mTK6 cells, expression of the functional *TK* allele is suppressed by DNA methylation (indicated by yellow circles) in the promoter region (*TK* ^m^). (A) mTK6 cells are cultured to logarithmic growth in the presence of TFT while maintaining DNA methylation-mediated suppression of the *TK* gene. (B) mTK6 cells are resuspended in fresh RPMI-1640 medium and exposed to the test compound or vehicle control for 24 h. (C) Following treatment of mTK6 cells with the test compound, cells are washed, adjusted to the appropriate density, plated onto revertant frequency (RF) and cloning efficiency (CE) plates with or without CHAT (2′-deoxyCytidine, hypoxanthine, aminopterin, and thymidine) selection, and incubated for 3 weeks for colony formation. (D) After 3 weeks of culture, colonies on CE and RF plates are counted, and TK revertant frequency, reflecting epigenetic changes in *TK* gene methylation status, is determined using Poisson distribution-based calculations.

## Background

A broad range of chemical substances has been implicated in carcinogenesis through genetic, epigenetic, and metabolic mechanisms. Chemical-induced genetic alterations, collectively referred to as genotoxicity, arise from direct DNA damage or from disruption to DNA replication, repair, and damage response pathways. To evaluate these risks, standardized in vitro genotoxicity assays are widely applied in accordance with the recommendations of the Organization for Economic Co-operation and Development guidelines [1]. However, increasing evidence indicates that many chemical agents contribute to carcinogenesis not only through the induction of DNA mutations but also via perturbation of epigenetic regulation, including changes in DNA methylation and histone modifications. Current chemical safety testing frameworks rely primarily on in vitro mutation-based assays, such as the Ames test and mammalian cell gene mutation assays [2-4], which are unable to assess epigenetic alterations. Although high-resolution sequencing-based epigenome profiling technologies can generate detailed information, their high cost, technical complexity, and significant analytical burden limit their routine application in regulatory testing, highlighting the need for practical approaches to assess epigenetic toxicity. To address this need, we developed a practical approach for assessing epigenetic alterations at a housekeeping gene locus by adapting the *TK* gene mutation assay [2,5], which utilizes the human lymphoblastoid TK6 cell line widely employed in genotoxicity testing. The *TK* gene mutation assay quantifies mutant frequency by measuring colony formation according to the *TK* genotype. Trifluorothymidine (TFT) is used to select TK-deficient mutant colonies because cells expressing functional thymidine kinase convert TFT into a toxic nucleotide and undergo cell death, whereas TK-deficient cells survive in the presence of TFT. Conversely, cells carrying a functional or reverted TK gene can be selectively detected using hypoxanthine–aminopterin–thymidine medium, in which only cells with restored thymidine kinase activity survive through the salvage pathway under aminopterin-mediated inhibition of de novo nucleotide synthesis. Building upon standardized in vitro genotoxicity testing frameworks, we developed the epi-TK assay, a mammalian cell–based reporter system in which the promoter region of the housekeeping *TK* gene in human lymphoblast TK6 cells is site-specifically methylated using CRISPR/dCas9-based epigenome-editing technology [6]. The engineered reporter cell line enables quantitative and bidirectional detection of chemically induced epigenetic effects through changes in frequency of TK revertant colony formation. Following chemical exposure, epigenetic alterations at the *TK* locus are assessed using hypoxanthine–aminopterin–thymidine selection, with restoration of *TK* gene expression quantified as the frequency of TK revertant colonies. Because the methylated *TK* locus retains active histone acetylation marks, it exists in a transcriptionally suppressed but reversible state, allowing spontaneous TK reactivation to occur at a measurable baseline frequency during culture [6]. An increase in revertant frequency suggests inhibition of DNA methylation or promotion of DNA demethylation, whereas a decreased reversion may reflect changes in histone modifications associated with reduced chromatin accessibility. These trends can be further validated by locus-specific DNA methylation analysis, chromatin immunoprecipitation, or protein-level assessment, depending on the expected mode of action of the test compound. Using GSK3484862 as a model DNA methyltransferase 1 inhibitor [7], this protocol quantitatively evaluates its epigenetic effects using the epi-TK assay. As previously reported, the assay also enables quantitative assessment of inflammation-associated global reductions in histone acetylation [6], further highlighting its bidirectional capability to detect distinct modes of epigenetic alteration. Collectively, this assay provides a simple, cost-effective, and biologically relevant platform for evaluating epigenetic toxicity and offers a practical approach for routine chemical safety assessment.

## Materials and reagents

**Biological materials**

1. Human lymphoblastoid mTK6 cell line (TK6-derived cell line originally established in a previous study [6]; available from the authors upon request), stored at -80 °C for short-term storage or in liquid nitrogen for long-term storage. Alternatively, equivalent cell lines can be established from the parental TK6 cell line or other cell types by isolating TFT-resistant clones generated via transient expression of dCas9-DNMT3A with guide RNAs targeting the *TK* gene promoter [6].

**Reagents**

1. RPMI-1640 medium (Nacalai Tesque, catalog number: 30264-56); store at 4 °C

2. Fetal bovine serum (FBS) (Nichirei Biosciences, catalog number: 174012-500ML); aliquot and store at -20 °C

3. 100 mM sodium pyruvate solution (Nacalai Tesque, catalog number: 06977-34); store at 4 °C

4. Penicillin-streptomycin solution (Nacalai Tesque, catalog number: 09367-34); store at 4 °C

5. Trifluorothymidine (TFT) (Sigma-Aldrich, catalog number: T2255-100MG)

6. 2′-Deoxycytidine hydrochloride (Sigma-Aldrich, catalog number: D0776)

7. Hypoxanthine (Sigma-Aldrich, catalog number: H9377)

8. Aminopterin Hybri-Max^TM^ (Sigma-Aldrich, catalog number: A5159)

9. Thymidine (Sigma-Aldrich, catalog number: T9250)

10. GSK-3484862 (CHEMIETEK, catalog number: CT-GSKMI)

11. Dimethyl sulfoxide (DMSO) (Nacalai Tesque, catalog number: 13408-64)

12. Hydrochloric acid (HCl) (Nacalai Tesque, catalog number: 18321-05)

**Solutions**

1. RPMI-1640 complete medium (see Recipes)

2. 1,000× TFT (see Recipes)

3. 1,000× 2′-deoxycytidine (see Recipes)

4. 1,000× hypoxanthine (see Recipes)

5. 1,000× thymidine (see Recipes)

6. 100× CHAT (2′-deoxyCytidine, hypoxanthine, aminopterin, and thymidine) (see Recipes)

**Recipes**

**1. RPMI-1640 complete medium**

**Table:** 

Reagent	Final concentration	Quantity or volume
RPMI-1640	n/a	500 mL
100 mM sodium pyruvate solution	1.8 mM	10 mL
Penicillin-Streptomycin mixed solution	88 units/mL	5.0 mL
Heat-inactivated FBS	8.8% v/v	50 mL

Store at 4 °C.

**2. 1,000× TFT**

**Table:** 

Reagent	Final concentration	Quantity or volume
Trifluorothymidine	3.0 mg/mL	15 mg
H_2_O	n/a	To 5 mL

Sterilize by filtration through a Minisart 0.2-μm syringe filter and store at -20 °C.

**3. 1,000× 2′-deoxycytidine**

**Table:** 

Reagent	Final concentration	Quantity or volume
2′-Deoxycytidine hydrochloride	10 mM	0.132 g
H_2_O	n/a	To 50 mL

Sterilize by filtration through a Minisart 0.2-μm syringe filter and store at -20 °C.

**4. 1,000× hypoxanthine**

**Table:** 

Reagent	Final concentration	Quantity or volume
Hypoxanthine	200 mM	1.361 g
1 M HCl	n/a	To 50 mL

Sterilize by filtration through a Minisart 0.2-μm syringe filter and store at -20 °C.

**5. 1,000× thymidine**

**Table:** 

Reagent	Final concentration	Quantity or volume
Thymidine	17.5 mM	0.2119 g
H_2_O	n/a	To 50 mL

Sterilize by filtration through a Minisart 0.2-μm syringe filter and store at -20 °C.

**6. 100× CHAT**

**Table:** 

Reagent	Final concentration	Quantity or volume
Aminopterin Hybri-Max^TM^	10 μM	1 vial (lyophilized powder)
H_2_O	n/a	14 mL
1,000× 2′-deoxycytidine	1 mM	2.0 mL
1,000× hypoxanthine	20 mM	2.0 mL
1,000× thymidine	1.75 mM	2.0 mL

Sterilize by filtration through a Minisart 0.2-μm syringe filter and store at -20 °C.

**Laboratory supplies**

1. 10-cm culture dish (AS ONE, catalog number: 1-7484-01)

2. 15-cm culture dish (SPL Life Science, catalog number: 20150)

3. 15- and 50-mL conical tubes (Greiner Bio-One, catalog numbers: 188271-N and 227261)

4. 1.5-mL microcentrifuge tube [Scientific Specialities Inc (SSI bio), catalog number: 1210-10]

5. Reagent reservoir (Greiner Bio-One, catalog number: J908305)

6. Serological pipettes (5, 10, 25, and 50 mL) (Greiner Bio-One, catalog numbers: 606180, 607180, 760180, and 768180)

7. 96-well flat-bottom plate (VIOLAMO, catalog number: 1-1610-06)

8. Minisart 0.2-μm syringe filter (Sartorius, catalog number: S7597FXOSK)

## Equipment

1. Pipetman P10, P20, P200, and P1000 (Gilson, catalog numbers: F144055M, F144056M, F144058M, and F144059M)

2. P200L multichannel micropipette (Gilson, catalog number: FA10011)

3. Pipet-Aid XP (Drummond, catalog number: 4-040-101-J)

4. Inverted microscope (Carl Zeiss, catalog number: Primovert)

5. Stereomicroscope (Leica, catalog number: M205 C)

6. Biological safety cabinet (Panasonic Healthcare, catalog number: MHE-S1301A2)

7. CO_2_ incubator (Panasonic Healthcare, catalog number: MCO-19AIC)

8. Multitube refrigerated centrifuge (TOMY SEIKO, catalog number: AX-511)

9. Beckman Coulter Z2 particle counter (Beckman Coulter, catalog number: 6605700)

10. Counting chamber (Erma, catalog number: 10-2170-05)

## Procedure

**A. Preparation of cell cultures**

*Note: All steps must be performed in a biological safety cabinet.*

1. Seed mTK6 cells at a density of 30,000 cells/mL in a 15-cm culture dish containing RPMI-1640 complete medium.

2. Add 1/1,000 volume of 1,000× TFT solution to the culture medium and incubate the cells for 3 days at 37 °C in a humidified CO_2_ incubator with 5% CO_2_.

*Note: TFT is added to the culture medium during initial culture to suppress the gradual accumulation of spontaneous TK revertants, thereby maintaining a stable baseline revertant frequency over serial passages.*

3. Examine cell morphology and assess contamination using an inverted microscope. Transfer the cell suspension to a 50-mL conical tube and determine the cell number using a Z2 particle counter or a counting chamber.

4. Calculate the appropriate dilution factor to adjust the cell density to 200,000–300,000 cells/mL. Seed the cells into a new 15-cm culture dish containing fresh RPMI-1640 complete medium and add 1,000× TFT solution at 1/1,000 of the final culture volume. Incubate the cells for an additional 24 h under the same conditions.

**B. Cell preparation for chemical exposure**

*Note: All steps must be performed in a biological safety cabinet.*

1. Transfer the entire culture volume to a 50-mL conical tube and determine cell density using a Z2 particle counter or counting chamber. At this stage, a cell density of approximately 800,000–1,000,000 cells/mL is recommended.

2. Based on the measured cell density, transfer a volume of the cell suspension corresponding to (number of treated groups + 1) × 2,500,000 cells into a new 50-mL conical tube.

3. Centrifuge the cells at 500× *g* for 5 min at 4 °C using a multitube cooling centrifuge. Carefully discard the supernatant containing RPMI-1640 complete medium with TFT and gently loosen the cell pellet by tapping.

4. Add fresh RPMI-1640 complete medium without TFT at a volume of (number of treated groups + 1) × 5 mL and resuspend the cells thoroughly.

5. Measure the cell density using a Z2 particle counter or a counting chamber and adjust the concentration to 500,000 cells/mL ± 5% (i.e., 475,000–525,000 cells/mL).

6. Aliquot 5.0 mL of the cell suspension into 15-mL conical tubes and add 4.9 mL of fresh RPMI-1640 complete medium to each tube. Add 100 μL of the test reagent (e.g., GSK3484862; final concentrations: 0.5, 2, or 5 μM) or 100 μL of DMSO as a vehicle control. Test reagent concentrations should be adjusted so that the cloning efficiency (CE, described below) of treated cells remains within approximately 20%–100% of the vehicle control, because cell proliferation during the treatment period is required to reveal epigenetic alterations. For compounds with unknown cytotoxicity, it is recommended to first evaluate a concentration range starting from 10 mM (or 2 mg/mL for compounds with molecular weight >200), typically using serial 10-fold dilutions, to identify concentrations suitable for the assay.

7. Transfer the contents of each 15-mL tube to 10-cm culture dishes by decanting and incubate the cells for 24 h at 37 °C in a humidified CO_2_ incubator with 5% CO_2_.

**C. Cell washing, cell density adjustment, and plating**

*Note: All steps must be performed in a biological safety cabinet.*

1. Transfer the entire cell suspension from each 10-cm dish into a 15-mL conical tube and centrifuge at 500× *g* for 5 min at 4 °C.

2. Discard the supernatant by decanting, gently loosen the cell pellet by tapping, add 5 mL of RPMI-1640 complete medium, mix by gentle inversion, and centrifuge at 500× *g* for 5 min at 4 °C.

3. Repeat step C2 once.

4. Discard the supernatant, gently loosen the cell pellet, resuspend the cells in RPMI-1640 complete medium, and determine the cell density using a Z2 particle counter or a counting chamber.

5. Based on the measured cell density, adjust each treatment group to a final concentration of approximately 200,000 cells/mL in a total volume of 10 mL using fresh RPMI-1640 complete medium.

6. Prepare serial dilutions of the cell suspension for plating on revertant frequency (RF) plates (two plates per treatment group) and cloning efficiency (CE) plates (one plate per treatment group), as shown in Tables 1 and 2 for the vehicle control and chemical treatment groups, respectively.

**Table 1.** Dilution scheme for vehicle control

Step	Purpose	Cell density (cells/mL)	Cell suspension	RPMI-1640 complete medium added	Total volume
0	Starting cell suspension	200,000	10 mL	-	10 mL
1	RF plates	5,000	1.25 mL (from step 0)	48.75 mL	50 mL
2	Intermediate dilution	200	0.40 mL (from step 1)	9.6 mL	10 mL
3	CE plate	8	1.0 mL (from step 2)	24 mL	25 mL

*Note: Cells from the vehicle control group are serially diluted from an initial concentration of 2.0 × 10^5^ cells/mL to prepare cell suspensions for revertant frequency (RF) plating at 5,000 cells/mL and cloning efficiency (CE) assays at 8 cells/mL.*

**Table 2.** Dilution scheme for the GSK3484862-treated groups

Step	Purpose	Cell density (cells/mL)	Cell suspension	RPMI-1640 complete medium added	Total volume
0	Starting cell suspension	200,000	10 mL	-	10 mL
1	Intermediate dilution	5,000	0.10 mL (from step 0)	3.9 mL	4.0 mL
2	RF plates	100	1.0 mL (from step 1)	49 mL	50 mL
3	CE plate	8	2.0 mL (from step 2)	23 mL	25 mL

*Note: For the GSK3484862-treated groups, cells are serially diluted from an initial concentration of 2.0 × 10^5^ cells/mL through an intermediate dilution step at 5,000 cells/mL to prepare cell suspensions for revertant frequency (RF) plating at 100 cells/mL and cloning efficiency (CE) plating at 8 cells/mL. For compounds with unknown epigenetic activity, it is recommended to preliminarily examine RF plating densities over a range of 100–100,000 cells/mL using serial 10-fold dilutions to identify conditions that yield interpretable colony numbers.*

7. After completing all dilution steps, add 100× CHAT solution at 1/100 of the final culture volume prepared for RF plating. In the present protocol, it corresponds to 500 μL for the vehicle control tube and 485 μL for the GSK3484862-treated tubes, based on their respective final suspension volumes shown in Tables 1 and 2.

8. Transfer the RF or CE cell suspensions to reagent reservoirs and seed 200 μL per well into 96-well flat-bottom plates using a P200L multichannel micropipette.

9. Incubate the plates for 3 weeks at 37 °C in a humidified CO_2_ incubator with 5% CO_2_.

**D. Colony counting and data analysis**

**Figure 1.**
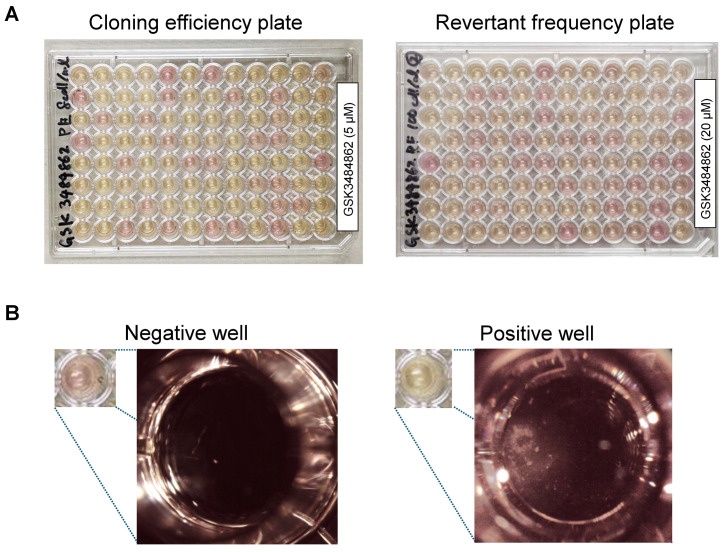
Representative images of cloning efficiency and revertant frequency plates generated in the epi-TK assay. (A) Representative images of cloning efficiency (CE) (left) and revertant frequency (RF) plates (right) prepared following chemical treatment. CE plates were used to determine cell survival, whereas RF plates were used to quantify revertant frequency. (B) Representative microscopic images of negative wells (no colony formation) and positive wells (visible colony formation). Wells were scored based on the presence or absence of colonies and used for Poisson-based calculation of CE and RF values.

1. Count the number of colonies on the CE and RF plates separately. First, place the plates on a white background and mark the wells in which the culture medium has changed color from red to yellow, indicating colony formation (Figure 1A).

2. Next, inspect each well directly under a stereomicroscope to confirm whether colonies are present in wells that were not marked in step D1. Colonies are observed as discrete cell aggregates within the wells, as shown in Figure 1B. Color change of the medium should not be used as the sole criterion for colony scoring and should be confirmed by microscopic observation to ensure accurate results.

3. Record the number of colonies for the CE and RF plates according to Tables 3 and 4.

4. Calculate CE using equation (1), based on the Poisson distribution [8], where EW represents the number of wells without colonies, TW represents the total number of wells, and N is the average number of cells per well (N = 1.6) in the CE plates (Table 3). Relative survival (%) is determined by comparing the CE values of chemically treated cells with those of the vehicle control.

CE = -ln (EW/TW)/N (1)

5. Calculate revertant frequency (RF) using equation (2), also based on the Poisson distribution, where N corresponds to the number of cells per well in the RF plates.

RF = [-ln (EW/TW)/N]/CE_treated_ (2)

**Table 3.** Cloning efficiency and relative survival of mTK6 cells following treatment with GSK-3484862

GSK-3484862 (μM)	Number of colonies (positive well)	Negative well	Total well	CE (%)	Relative survival
0 (vehicle control)	75	21	96	95	100
0.5	69	27	96	79	83
2.0	60	36	96	61	65
5.0	54	42	96	52	54

**Table 4.** TK revertant frequency of mTK6 cells following treatment with GSK-3484862

GSK-3484862 (μM)	Number of colonies (positive well)		Total positive well	Negative well	Total well	RF
	**Plate 1**	**Plate 2**				
0 (Vehicle control)	20	15	35	157	192	2.1 × 10^-4^
0.5	56	54	110	82	192	5.4 × 10^-2^
2.0	65	68	133	59	192	9.6 × 10^-2^
5.0	57	65	122	70	192	9.8 × 10^-2^

**Figure 2.**
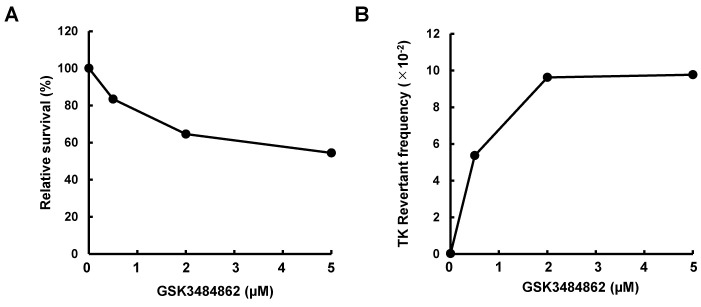
Relative cell survival and thymidine kinase (TK) revertant frequency measured using the epi-TK assay following GSK3484862 treatment. Cytotoxic and epigenetic effects were measured as relative cell survival (A) and TK revertant frequency (B), respectively, following treatment with GSK-3484862. The data are representative of a single independent experiment.

6. Plot the results by displaying chemical concentration on the x-axis and relative survival and TK revertant frequency on the y-axis, as shown in Figure 2A, B.

7. Repeat all experiments independently at least three times and statistically analyze the data using Dunnett’s test by comparing chemical-treated groups with the vehicle control.

## Validation of protocol

This protocol has been applied and validated in the following research article:

- Yamada et al. [6]. Dual-directional epi-genotoxicity assay for assessing chemically induced epigenetic effects utilizing the housekeeping *TK* gene. *Scientific reports* (Figure 2).

## General notes and troubleshooting

**General notes**

1. In this protocol, CHAT medium is used for selection. Although hypoxanthine–aminopterin–thymidine medium supports selection of TK revertants, CHAT provides more stable colony formation and improved reproducibility by supplementing pyrimidine metabolism with cytidine.

2. Spontaneous revertant frequency may vary slightly depending on the solvent used; therefore, a vehicle control should be included for each test chemical.

3. For chemical-treated groups, the RF plating density **should be optimized** based on the anticipated epigenetic effects of the test compound. Compounds that promote DNA methylation, inhibit demethylation, or enhance heterochromatin formation **may require higher RF plating densities** than the vehicle control. In contrast, compounds that inhibit DNA methylation, promote demethylation, or enhance euchromatin formation **may require lower RF cell densities** than the vehicle control. The plating density used in this protocol was selected to obtain approximately 10–70 colonies per 96-well plate under baseline conditions, which provides reliable revertant frequency calculation based on the Poisson distribution. This density also allows stable detection of both increased revertant frequency induced by DNA demethylating agents and reduced colony formation caused by compounds affecting chromatin regulation.

4. Chemical treatment conditions that result in excessive cytotoxicity impair cell proliferation and may compromise RF colony formation. Because cell proliferation during the treatment period is required to reveal epigenetic alterations, test compound concentrations should be selected to maintain a relative CE of at least 20% compared with the vehicle control. As a preliminary optimization step, cells may be washed to remove the compound after treatment and cultured for an additional 24 h, after which cell proliferation can be assessed.

5. Horse serum (HS) can be used as an alternative to FBS. The RF colony formation typically proceeds more slowly in HS- supplemented cultures than in FBS-supplemented cultures; however, an incubation period of up to 3 weeks is sufficient to allow visible colony formation under either condition.

6. When metabolic activation is required (e.g., through the use of an S9 mix), the chemical treatment duration should be reduced from 24 to 4 h. Following treatment, cells should be washed to remove both the test compound and the S9 mix, followed by a 24-h recovery period before proceeding with CE and RF plating.

7. Our previous findings indicate that the methylated *TK* locus is enriched in histone acetylation marks and therefore appears unresponsive to histone deacetylase inhibitors that generally induce chromatin relaxation [6]. In contrast, compounds causing global reduction of histone acetylation produce measurable changes in TK revertant frequency, supporting the utility of this assay for detecting such epigenetic effects.

**Troubleshooting**

**Problem 1**: Fungal contamination is observed in some wells of the CE or RF plates.

Possible cause: Due to the extended incubation period (up to 3 weeks), fungal contamination may occur depending on CO_2_ incubator conditions.

Solution: If fungal contamination is detected, exclude the affected wells from analysis and calculate CE and RF values using only uncontaminated wells.

**Problem 2**: Colonies are observed in all wells of the RF plates.

Possible cause: CHAT selection pressure is insufficient to restrict colony formation to TK revertant cells. Alternatively, the cell density for plating is inappropriate for detecting the epigenetic effects of the test compound.

Solution: Aminopterin is available from multiple suppliers and in various grades; the performance of alternative products has not been validated. Prepare CHAT strictly according to the protocol described in the Recipes section. In addition, optimize the RF plating density within a range of 100–100,000 cells/mL, depending on the expected epigenetic mode of action of the test compound.
